# Item analysis of university-wide multiple choice objective examinations: the experience of a Nigerian private university

**DOI:** 10.1007/s11135-017-0499-2

**Published:** 2017-03-14

**Authors:** Jonathan A. Odukoya, Olajide Adekeye, Angie O. Igbinoba, A. Afolabi

**Affiliations:** Covenant University, Ota, Nigeria

**Keywords:** Item analysis, Multiple choice questions, Examination, Dificulty Index

## Abstract

Teachers and Students worldwide often dance to the tune of tests and examinations. Assessments are powerful tools for catalyzing the achievement of educational goals, especially if done rightly. One of the tools for ‘doing it rightly’ is item analysis. The core objectives for this study, therefore, were: ascertaining the item difficulty and distractive indices of the university wide courses. A range of 112–1956 undergraduate students participated in this study. With the use of secondary data, the ex-post facto design was adopted for this project. In virtually all cases, majority of the items (ranging between 65% and 97% of the 70 items fielded in each course) did not meet psychometric standard in terms of difficulty and distractive indices and consequently needed to be moderated or deleted. Considering the importance of these courses, the need to apply item analyses when developing these tests was emphasized.

## Introduction

Multiple choice objective tests items are easy to score and analyze but often technical, time consuming and at times painstaking in development. To cover a wide scheme of work or syllabus adequately, it is imperative that multiple-choice objective test be used. When assessing a large population of students, the use of multiple-choice question (MCQ) is the most logical option. The challenges however are: tendency to write poor MCQs with ambiguous prompts, poor distractors, multiple answers when question demands only one correct answer, controversial answers, give-away keys, higher probability of testees guessing correctly to mention but few of the challenges of developing and using MCQs.

There is hardly any subject that cannot use MCQ. However, when assessments border on life sensitive issues like health, air flight (and the like), it should be applied with caution. The reality, however, is that virtually all assessment purposes are life sensitive. The results of virtually all assessments are often used to make sensitive decision that determine people’s destiny. It is therefore imperative that MCQs be handled rightly at the development, administration, scoring, grading and interpretation stages. The focus of the study reported here is on the development stage of MCQs, with particular emphasis on item analyses.

The first critical step in developing valid MCQs is recruiting relevant subject experts with requisite skill in writing of MCQ items. The correct handling of this stage will go a long way in setting the pace for the establishment of the content validity of the test. However, the validity of MCQs cannot be completely ascertained with skillful item writing alone. Psychometric requirement demands that such items be trial tested, while the responses and scores generated are subjected to statistical item analyses.

Ary et al. (2002) opined that item analysis involves use of statistics that can provide relevant information for improving the quality and accuracy of multiple choice question. There are three popular forms of item analyses: *item difficulty index, distractive index* and *discriminatory index*.

*Item difficulty index* indicates the degree of difficulty of the MCQ items in relation to the cognitive ability of the testees (Boopathiraj and Chellamani 2013). It is calculated by finding the proportion of the testees that got the item correctly. An item is adjudged too difficult when the index is below 0.3. An item is adjudged too easy when the index is above 0.7. Depending on the purpose of the test, the cut off points for easy or difficult items can be adjusted upward or downward. Generally, the rule is that life sensitive or competitive activities require more technical/difficult items in screening; while less sensitive activities or activities requiring motivation of testees often use less difficult items. For most summative assessments, such as those handled by the West African Examinations Council, moderate difficulty index ranging around 0.5 are often preferred.

It is important to note that an item may record high difficulty index if the content of such item was not taught, the concept was not understood or if the question was not properly worded. According to Suruchi and Rana (2015) the two purposes of Item analysis are: firstly, to identify defective test items and secondly, to indicate the areas where the learners have or have not mastered. This is actually the essence of item analysis—to check for flaws of this nature and find ways of correcting them before finally administering the questions (El-Uri and Malas 2013). Item moderation, therefore, naturally follows item analysis. Where an item cannot be moderated, it is often discarded and replaced.

The distractive index determines the power of the distractor (i.e. the incorrect options in a MCQ) in distracting the testees. The distractive index is computed in virtually the same way as the difficulty index. It is the proportion of tesstes who selected a distractor out of all the testees that sat for the test. When a distractor distracts few or no testee, it is concluded that such is a poor distractor and should be reviewed. When a distractor over-distracts, that is, distracts about the same proportion or higher proportion of the testees that are selecting the key (i.e. right option), such option is also due for review or replacement.

Sabri (2013) submitted that *discriminatory index* depicts the power of an item in discriminating between high and low performing Testees. Item discrimination determines whether those who did well on the entire test did well on a particular item. An item should in fact be able to discriminate between upper and lower scoring groups. One way to determine an item’s power to discriminate is to compare those who have done very well with those who have done very poorly, known as the extreme group method. First, identify the Testees who scored in the top one-quarter (upper quartile) as well as those in the bottom one-quarter of the class (lowest quartile). Next, calculate the proportion in the upper and lower quartiles that answered a particular test item correctly. Finally, subtract the proportion of Testees who got the item right in the bottom performing group from the proportion of Testees in the top performing group who got the item right to obtain the item’s discrimination index (D). Item discriminations of D = 0.50 or higher are considered excellent. D = 0 means the item has no discriminatory power, while D = 1.00 means the item has perfect discrimination power. It is therefore expected that more of the high performing Testees should get an item right while few of the low performing students should get the same item right. When more Testees who generally perform poorly in a test tend to select the right option for an item and those who performed well are selecting wrong options as answer, then something is apparently wrong with such an item. It calls for item review or discard. Thus, item analyses activities work to enhance the overall validity of a test.

Kehoe (1995) observed that the basic idea that we can capitalize on is that the statistical behavior of “bad” items is fundamentally different from that of “good” items. This fact underscores the point of view that tests can be improved by maintaining and developing a pool of “good” items from which future tests can be drawn in part or in whole. This is particularly true for instructors who teach the same course more than once. Item analysis is a tool to help the item writer improve an item (Gochyyev and Sabers 2010).

Over the years, tertiary institutions have come to realize the significance of some life-enhancing concepts that should be learnt. It is these vital life-enhancing information that have been packaged as university wide courses. Consequently, some universities have compulsory courses like General Studies, which covers use of languages and philosophical issues; Total Man Concept; Entrepreneurship Development Studies; Human Development etc. Some of these courses are zero unit but compulsory. The truth is that knowledge, especially applicable and relevant knowledge, are powerful and life transforming. It is therefore imperative to teach and assess these courses professionally for maximum impact. It is against the backdrop of these points this study was undertaken.

### Statement of problem

Inadvertent omission of item analysis in the process of developing Multiple Choice Questions (MCQ) for compulsory university-wide courses that solely use MCQ could jeopardize the integrity of assessment and certification. Incorrect application of item analysis results could yield the same fate. As a compulsory course, failure could translate to affected students spending extra year on campus. This has implications on the psychological state of concerned students’. The emotional offshoot of failing and having to spend an extra year with one’s juniors could translate to a number of debilitating medical, psychosomatic and psychological challenges. On the other hand, unprofessional assessment could lead to wrong award of grades and certificates. In the study of computer adaptive testing, Cechova et al. (2014) reiterated this point when they surmised: ‘Every year, hundreds of secondary school students take university entrance exams, and their results determine entry into universities or possible alternatives, such as employment. In the same way, every year university teachers face the challenge of how to cope with the increasing number of examination candidates vis-à-vis maintaining the validity of the tests’.

### Statement of significance

Professional conduct of item analysis and concomitant item moderation of items comprising the university wide courses is apt to enhance the overall validity of such tests. This in turn is apt to significantly reduce frustrations for the individual and the society at large. Correct assessment, with application of essential psychometric practices like item analysis is apt to enhance the quality of assessment, evaluation and certification (IAR, 2011).

### Statement of objectives

- Find out how appropriate the difficulty indices of the items comprising the university wide courses are?
- Determine the appropriateness of the distractive indices of the options making up the items in the university-wide course MCQs?

### Research questions

- How appropriate are the difficulty indices of the items comprising the university wide courses?
- How appropriate are the distractive indices of the options making up the items in the university wide course MCQs?

## Method

The ex-post facto design was adopted for this study. Secondary data were collated and analyzed.

The population for this study were undergraduates of private universities in Nigeria. They were estimated at about one million as at the time of this study.

The responses of over 1500 students that responded to the MCQs of the university-wide courses at various times were harvested and analyzed. Students responses in following courses were analyzed: EXX 121 (N = 1907; Test taken 2015); GXX 121; N = 1956; Test taken 2015); HXX 421 (N = 112; Test taken 2015); TXX 121 (N = 1905; Test taken 2015). Note that original course codes have been changed for anonymity. These courses were chosen for this study largely because they are compulsory for students’ graduation, irrespective of program, and because the relevance of the course content to overall wellbeing cum success in life.

The core *instruments* for this study were the past MCQ items for four core university-wide courses.

The responses to past MCQs were harvested from the University’s Data Centre.

The major statistical analyses conducted were difficulty index and distractor index, using proportion and simple percentage. The formulas applied in this regard are:

$${\text{Distractive}}\,{\text{index}} = \frac{Number\,of\,times\,an\,option\,was\,selected}{Total\,number\,of\,respondents}$$

For multiple choice questions with one correct answer format:

$${\text{Difficulty}}\,{\text{index}} = \frac{Number\,of\,respondents\,who\,selected\,the\,right\,option}{Total\,number\,of\,respondents}$$

The following decision rules were applied to determine items that are Okay (OK), Fairly Okay (F/OK), Need Moderation (NM), and Need Serious Moderation (NSM): When the difficulty index is over 0.7 (i.e. 70%) or below 0.2 (i.e. 20%), such item is adjudged not okay and needs moderation. The difficulty index was computed with the proportion of Testees selecting the correct option as indicated by the bold figures in Table 1 below. When the distractive index for a distractor or incorrect option is far above or far below 0.166 (i.e. 16.6%), there is need for moderation. The rationale for this decision is that for a test that operates by the principle of moderate difficulty of 0.5, the remaining 0.5 should be fairly shared equally between the 3 distractors (for a 4-option item), which gives 0.166. Any item falling short of these two requirements is apt to require moderation.

**Table 1:** 2015 EXX 121 N = 1907

Items	A (%)	B (%)	C (%)	D (%)	E (%)	Comment
1	1.2	0.5	5.1	16.6	**76.5**	Need serious moderation (NSM)
2	16.3	5.3	20.8	**35.7**	21.4	Need moderation (NM)
3	**72.8**	1.9	6.7	17.6	0.5	NSM
4	**90.0**	9.4	0.1	0.1	0.1	NSM
5	**37.3**	60.8	0.1	0.7	0.2	NSM
6	6.4	9.6	**17.2**	53.3	12.7	NSM
7	**15.7**	82.7	0.3	0.7	0.1	NSM
8	**30.3**	8.4	4.2	34.9	21.6	NSM
9	**80.0**	18.8	0.3	0.4		NSM
10	64.3	**26.4**	2.7	5.2	0.2	NSM
11	**94.6**	3.0	2.2	0.1	0.1	NSM
12	**94.3**	4.7	0.4	0.1		NSM
13	4.6	**16.9**	35.1	42.6	0.2	NSM
14	**22.5**	1.6	2.8	1.9	71.1	NSM
15	**83.6**	15.5	0.1	0.2	0.1	NSM
16	**79.1**	19.9	0.2	0.2	0.2	NSM
17	1.5	1.4	9.0	41.6	**46.1**	NM
18	0.4	0.8	21.8	53.8	**22.9**	NSM
19	19.0	4.3	6.1	**62.7**	7.0	NM
20	11.6	48.9	13.6	**24.0**	0.6	NSM
21	20.5	14.7	**47.2**	16.7	0.4	OK
22	6.4	**20.5**	3.3	1.2	67.6	NSM
23	73.6	1.0	**24.5**	0.5	0.2	NSM
24	0.5	12.8	3.3	**81.5**	1.5	NM
25	**82.1**	16.3	0.3	0.3		NM
26	11.7	**49.7**	20.1	17.0		OK
27	**94.1**	5.2	0.2	0.1		NSM
28	**11.5**	19.1	18.2	47.4	3.1	NSM
29	**92.3**	6.6	0.2	0.2		NSM
30	40.5	**58.4**	0.2	0.4	0.2	F/OK
31	**67.1**	2.0	2.3	9.1	18.9	NM
32	1.7	6.2	43.1	21.2	**27.1**	NSM
33	**86.6**	12.6	0.3	0.1	0.2	NSM
34	12.0	11.1	13.6	3.3	**59.1**	F/OK
35	8.0	12.7	**64.9**	13.7	0.4	F/OK
36	23.7	23.6	23.0	**28.8**	0.1	NSM
37	**99.0**	0.8	0.1	0.1		NSM
38	42.7	**56.7**	0.1	0.2		NM
39	**88.4**	11.2	0.2	0.1	0.1	NSM
40	**92.6**	6.7	0.2	0.2		NSM
41	9.4	13.7	**75.4**	0.8		NM
42	**78.8**	15.6	3.5	1.7	0.1	NSM
43	14.1	**80.1**	1.0	4.3	0.1	NSM
44	**50.1**	13.6	3.6	32.2	0.1	OK
45	14.3	22.4	**41.6**	7.7	13.2	OK
46	12.5	22.5	25.6	19.9	**18.5**	NSM
47	**72.1**	3.8	5.9	10.5	7.3	NM
48	9.2	5.6	**10.4**	15.9	58.5	NSM
49	5.5	21.6	5.3	4.4	62.5	F/OK
50	31.0	38.5	27.7	2.3	**0.1**	NSM
51	**92.8**	1.6	0.6	4.7		NSM
52	3.0	94.2	1.1	**1.5**		NSM
53	39.7	15.6	26.1	**17.1**	0.1	NSM
54	**20.5**	51.7	15.8	10.5	0.1	NSM
55	**80.9**	3.7	10.0	4.7		NM
56	**42.2**	26.0	23.8	6.9	0.2	F/OK
57	26.7	58.0	**3.8**	10.5	0.1	NSM
58	12.5	**1.6**	1.5	83.5	0.1	NSM
59	**1.8**	3.0	88.9	5.4	0.2	NSM
60	70.1	**11.5**	6.6	10.3	0.3	NSM
61	23.3	**49.6**	23.6	3.1		F/OK
62	**0.8**	10.0	5.0	84.0		NSM
63	**6.0**	0.4	1.2	92.2	0.2	NSM
64	72.8	6.0	6.0	**10.4**	0.2	NSM
65	**7.7**	79.8	4.0	2.4	0.2	NSM
66	30.7	7.9	4.5	35.2	**21.1**	NSM
67	14.4	**29.7**	8.1	11.6	35.6	NSM
68	1.9	**9.1**	30.8	56.9	0.5	NSM
69	11.9	**5.9**	56.2	24.3	0.3	NSM
70	3.7	1.4	3.8	56.3	**34.6**	NM

Bold values are the indices for the correct options, which also represent the ‘difficulty indices’

## Results

The item analysis results in Tables 1 and 2 show that majority of the items (approximately 86% of the 70 items fielded) did not meet psychometric standard (of appropriate difficulty and distractive index) and consequently need moderation.

**Table 2:** Summary for 2015 EXX 121 (1907 Students)

Description	Frequency (N = 70)	%
1. Items that are *okay*	4	5.7
2. Items that are *fairly okay*	6	8.6
3. Items that *need moderation (NM)*	11	15.7
4. Items that *need serious moderation (NSM)*	49	70

The detailed table of results from which the summary in Table 3 was drawn is in “Appendix 1”. The item analysis results in Table 3 show that a notable proportion of the items (approximately 66% of the 70 items fielded) did not meet psychometric standard (of appropriate difficulty and distractive index) and consequently require moderation or deletion.

**Table 3:** Summary for 2015 GXX 121 (1956 Students)

Description	Frequency (N = 70 items)	%
1. Items that are *okay*	8	11.4
2. Items that are *fairly okay*	16	22.9
3. Items that *need moderation (NM)*	12	17.1
4. Items that *need serious moderation (NSM)*	34	48.6

The detailed table of results from which the summary in Table 4 was drawn is in “Appendix 2”. The item analysis results in Table 4 show that a notable proportion of the items (approximately 97.1% of the 70 items fielded) did not meet psychometric standard (of appropriate difficulty and distractive index) and consequently need moderation. Only 2.9% of the items were fairly okay. The operational psychometric standard for this study is that at least 70% of the items should be *okay* while the remaining 30% could be *fairly okay*.

**Table 4:** Summary for 2015 HXX 421 (112 Students)

Description of items	Frequency (N = 70 items)	%
1. Items that are *okay*	–	
2. Items that are *fairly okay*	2	2.9
3. Items that *need moderation(NM)*	15	21.4
4. Items that *need serious moderation (NSM)*	53	75.7

The detailed table of results from which the summary in Table 5 was drawn is in “Appendix 3”. The item analysis results in Table 5 show that a significant majority of the items (approximately 83% of the 70 items fielded) did not meet psychometric standard (of appropriate difficulty and distractive index) and consequently need moderation.

**Table 5:** Summary for 2015 TXX 121 (1905 Students)

Description of items	Frequency (N = 70 items)	%
1. Items that are *okay*	2	2.9
2. Items that are *fairly okay*	10	14.3
3. Items that *need moderation (NM)*	11	15.7
4. Items that *need serious moderation (NSM)*	47	67.1

## Discussion

The core research questions for this study are: ‘*How appropriate are the difficulty indices of the items comprising the university wide courses*?’; and ‘*How appropriate are the distractive indices of the options making up the items in the university wide course MCQs*?’ Results displayed in Tables 1, 2, 3, 4 and 5 and in Appendices 1, 2 and 3 show that the sampled university-wide courses (EXX 121, GXX 121, HXX 421, TXX 121) were not appropriate in terms of difficulty and distractive indices. In virtually all cases, majority of the items (ranging between 65% and 97% of the 70 items fielded in each course) did not meet the psychometric standard used in this study. These findings call for concern.

Contrary to the core findings in this study, Bichi (2015) found that out of the 40 items in a test assessed, 12 (30%) items failed to meet the set criteria of item quality and therefore needed moderation while 28 items were judged to be ‘good’ items. This appears to be a better result, yet he further recommended that the assessment of science secondary school students’ achievement should be subjected to item analysis to improve their quality.

In a related post-examination analysis of objective tests, Tavakol and Dennick (2011) reiterated that one of the key goals of assessment in medical education is the minimization of all errors influencing a test in order to produce an observed score which approaches a learner’s ‘true score’, as reliably and validly as possible. This is actually the core objective of all empirical assessment worldwide. From the results obtained from the current study therefore, it may be difficult to unequivocally conclude that the scores obtained by the students were their true scores and a true reflection of their ability. There is clearly need to conduct further psychometric assessment of these and related courses to ascertain the veracity of these findings.

## Recommendations and conclusion

This study sought to establish appropriateness of the difficulty and distractive indices of four compulsory university-wide courses in a Nigerian private university. In virtually all cases, majority of the items (ranging between 65% and 97% of the 70 items fielded in each course) did not meet psychometric standard in terms of difficulty and distractive indices. On the strength of the findings made from this study, and based on recent submissions on this subject (as cited above), it is recommended that the development of all the university-wide courses employing the MCQ format should commence with preparation of test blueprint followed by carefully adherence with the rules for writing multiple-choice objective questions (MCQs). Thereafter all items should be trial tested, item analyzed and subjected to item moderation to enhance the overall *content* and *construct* validities. This exercise will require the input of subject and psychometric experts. The exercise should be part of statutory quality assurance procedures. Dogged adoption of this singular recommendation is apt to significantly enhance the quality of graduates and certification in higher institutions.

## Appendix 1

See Table 6.

**Table 6:** GXX 121 full table of results

ITEMS	A (%)	B (%)	C (%)	D (%)	E (%)	Blank	Multiple
1	46.5	**35.2**	10.7	6.6		1.0	
2	1.7	7.5	7.8	**82.8**	0.1	0.2	
3	2.6	8.1	**80.4**	8.1		0.7	0.1
4	**48.6**	1.2	14.6	34.8	0.1	0.5	0.2
5	15.3	12.5	**61.9**	9.4		0.7	0.2
6	37.0	11.3	17.6	**32.5**		1.5	0.1
7	33.2	15.1	**25.1**	26.0		0.6	0.1
8	6.3	21.2	**61.7**	10.0		0.8	0.1
9	**41.9**	10.7	19.6	25.9	0.1	1.7	0.2
10	**31.3**	33.0	21.8	11.1		2.6	0.2
11	**32.2**	5.0	3.1	58.9		0.6	0.2
12	4.7	5.7	**50.3**	38.3		1.0	
13	4.4	**86.0**	0.7	8.8		0.1	
14	**71.4**	26.0	0.6	0.9		1.0	0.1
15	21.7	**63.7**	2.9	11.1		0.6	0.1
16	**37.2**	13.5	18.8	28.1	0.1	2.3	
17	5.5	41.4	**42.8**	9.8		0.5	0.1
18	**36.6**	52.6	7.2	2.6		1.1	0.1
19	15.4	2.1	50.7	**31.2**		0.4	0.1
20	17.2	**5.8**	75.4	1.2		0.3	0.1
21	1.9	2.5	**89.3**	6.1		0.2	
22	18.6	**50.6**	10.0	19.7	0.1	0.9	0.2
23	**27.9**	60.7	5.5	4.8		1.0	0.1
24	4.7	**68.0**	25.8	0.7		0.9	0.1
25	**55.8**	2.9	25.9	14.1	0.1	1.4	
26	11.1	**71.4**	2.1	14.4	0.1	1.0	
27	1.4	3.3	**73.0**	21.6	0.1	0.6	0.1
28	4.2	4.1	2.4	**88.7**	0.1	0.5	0.1
29	48.9	21.9	4.9	**23.5**		0.8	0.1
30	**57.8**	4.3	25.6	11.9		0.4	0.1
31	29.8	**33.1**	16.0	20.2	0.1	0.8	0.2
32	5.9	39.2	**36.5**	17.1		1.2	0.1
33	18.4	13.8	**63.7**	3.1		1.1	0.1
34	14.5	34.7	8.0	**41.2**		1.5	0.1
35	1.1	17.2	6.6	**74.6**	0.1	0.5	
36	45.0	15.1	10.7	**27.1**	0.1	1.9	0.1
37	13.7	**26.6**	6.4	52.0	0.1	1.2	0.1
38	53.0	4.9	15.3	**26.2**		0.5	0.1
39	**57.1**	12.9	23.5	5.4		1.1	0.1
40	12.4	**77.5**	5.4	4.0	0.1	0.7	
41	37.7	23.5	**35.6**	2.2		0.8	0.2
42	10.4	**74.6**	9.4	5.3		0.3	0.1
43	18.1	21.5	20.0	**39.3**	0.1	1.0	
44	**30.5**	28.9	32.6	6.6	0.1	1.2	0.1
45	8.0	7.3	**58.2**	24.9	0.1	1.5	0.1
46	26.5	**20.8**	22.5	28.7	0.1	1.2	0.2
47	37.8	23.1	**9.7**	27.6	0.1	1.7	
48	27.2	13.5	12.4	**46.1**		0.7	
49	**30.6**	24.8	17.3	25.3	0.1	1.9	0.1
50	11.6	**32.3**	37.2	17.6	0.1	1.3	
51	32.2	11.0	**45.4**	10.4		0.8	0.2
52	71.1	**18.1**	4.1	5.6		1.1	0.1
53	6.9	6.7	25.5	**60.0**		0.9	0.2
54	**49.8**	11.8	24.5	12.8		1.0	0.1
55	11.4	**45.8**	11.1	30.0	0.1	1.5	0.1
56	13.3	8.2	27.6	**49.0**		1.9	0.1
57	18.9	6.6	**50.6**	22.6	0.1	1.2	0.1
58	19.8	**16.5**	27.5	34.5		1.6	0.1
59	29.6	8.6	19.0	**41.1**		1.6	0.1
60	17.4	14.8	18.1	**47.5**		2.0	0.1
61	**39.8**	35.7	9.5	14.5	0.1	0.5	
62	**54.4**	18.1	22.0	4.7		0.8	
63	8.0	**47.4**	36.6	7.0	0.1	0.9	0.1
64	31.8	26.7	12.9	**27.5**		1.1	0.1
65	**51.4**	12.4	21.6	13.5		1.0	0.1
66	10.9	17.5	**66.6**	3.8		1.1	
67	23.6	21.5	24.1	**29.5**		1.4	
68	18.2	**15.3**	17.0	48.2	0.3	1.0	0.1
69	38.4	32.2	10.2	**18.0**	0.1	1.0	
70	30.0	18.4	**17.1**	32.8	0.7	1.0	0.1

Bold values are the indices for the correct options, which also represent the ‘difficulty indices’

## Appendix 2

See Table 7.

**Table 7:** HXX 421 full table of results

Items	A (%)	B (%)	C (%)	D (%)	E (%)	Blank
1	50.9%	**24.1%**	15.2%	8.9%	–	0.9%
2	3.6	**89.3**	1.8	3.6		1.8
3	3.6	–	**86.6**	1.8	8.0	
4	47.3	**51.8**	0.9	–	–	
5	3.6	25.0		**0.9**	69.6	0.9
6	8.9	8.9	20.5	**60.7**	0.9	
7	0.9	5.4	2.7	**87.5**	3.6	
8	24.1	4.5	**69.6**	0.9	0.9	
9	**8.9**	22.3	7.1	15.2	46.4	
10	–	–	**78.6**	5.4	16.1	
11	8.0	–	2.7	18.8	**69.6**	0.9
12	6.3	16.1	**68.8**	7.1	0.9	0.9
13	4.5	**86.6**	1.8	6.3	0.9	
14	5.4	9.8	4.5	**77.7**	2.7	
15	0.9	0.9	**97.3**	–	–	0.9
16	–	**100.0**	–	–	–	
17	**55.4**	2.7	–	40.2	1.8	
18	6.3	0.9	**88.4**	4.5		
19	**91.1**	2.7	1.8	2.7	0.9	0.9
20	5.4	2.7	3.6	**88.4**	–	
21	2.7	0.9	7.1	**89.3**	–	
22	–	0.9	**76.8**	16.1	6.3	
23	–	62.5	1.8	**5.4**	30.4	
24	9.8	0.9	11.6	1.8	**75.9**	
25	63.4	33.9	**2.7**	–	–	
26	6.3	23.2	–	1.8	**68.8**	
27	**91.1**	–	7.1	0.9	–	0.9
28	0.9	1.8	–	**95.5**	1.8	–
29	**97.3**	1.8	–	–	–	0.9
30	**18.8**	61.6	17.0	–	2.7	–
31	–	**96.4**	3.6	–	–	–
32	–	12.5	87.5	–	–	–
33	8.0	44.6	–	**47.3**	–	–
34	31.3	–	17.9	50.9	–	–
35	**71.4**	25.0	–	–	1.8	1.8
36	1.8	0.9	3.6	**58.0**	35.7	–
37	–	1.8	**97.3**	–	–	0.9
38	**85.7**	3.6	8.9	0.9	–	0.9
39	15.2	1.8	2.7	**80.4**	–	
40	**67.0**	25.9	7.1	–	–	
41	0.9	–	**9.8**	–	89.3	
42	–	**99.1**	–	0.9	–	
43	0.9	**98.2**	–	0.9	–	
44	–	97.3	–	2.7	–	
45	8.0	**6.3**	9.8	75.0	–	0.9
46	**65.2**	5.4	28.6	–	–	0.9
47	0.9	1.8	**95.5**	1.8	–	
48	48.2	15.2	3.6	**32.1**	–	0.9
49	5.4	**89.3**		4.5	–	0.9
50	0.9	16.1	8.0	**75.0**	–	
51	16.1	82.1	–	**0.9**	–	0.9
52	–	**99.1**	–	–	–	0.9
53	1.8	–	12.5	**85.7**	–	
54	15.2	54.5	2.7	**25.9**	–	1.8
55	**100.0**	–	–	–	–	
56	–	24.1	**73.2**	1.8	–	0.9
57	–	–	1.8	**98.2**	–	
58	21.4	1.8	–	70.5	**4.5**	1.8
59	–	5.4	**90.2**	1.8	1.8	0.9
60	–	**77.7**	0.9	16.1	4.5	0.9
61	**9.8**	84.8	1.8	2.7	–	0.9
62	**92.0**	0.9	5.4	0.9	0.9	
63	**79.5**	7.1	8.9	0.9	2.7	0.9
64	–	0.9	–	0.9	**98.2**	
65	0.9	–	5.4	19.6	**74.1**	
66	9.8	**47.3**	–	–	42.9	
67	17.9	**72.3**	2.7	4.5	1.8	0.9
68	–	33.9	**62.5**	3.6	–	
69	20.5	**50.0**	1.8	25.9	0.9	0.9
70	75.0	**6.3**	17.0	–	0.9	0.9

Bold values are the indices for the correct options, which also represent the ‘difficulty indices’

## Appendix 3

See Table 8.

**Table 8:** TXX 121 full table of results

Items	A (%)	B (%)	C (%)	D (%)	Blank	Multiple
1	1.3	7.0	**90.4**	1.0	0.3	
2	14.1	4.8	**72.6**	7.6	0.9	
3	67.4	18.5	**12.8**	0.5	0.8	
4	33.3	**32.0**	10.7	22.4	1.5	
5	10.2	**89.1**	0.2	0.2	0.3	
6	1.9	3.6	**66.4**	27.4	0.7	
7	1.4	**78.6**	17.5	1.9	0.6	
8	10.2	**89.1**	0.2	0.2	0.3	
9	6.4	**72.5**	17.7	2.1	1.2	0.1
10	1.9	**97.6**	0.1	0.1	0.3	
11	10.7	11.4	8.7	**68.6**	0.6	0.1
12	**82.5**	2.8	8.0	5.4	1.2	
13	23.1	**29.5**	20.9	24.6	1.8	0.1
14	**98.9**	0.9		0.1	0.2	
15	0.9	**97.3**	1.4	0.2	0.3	
16	12.9	**81.2**	0.7	4.8	0.4	
17	**77.6**	22.0		0.1	0.3	
18	0.8	1.8	**96.5**	0.6	0.3	
19	5.1	11.1	13.5	**69.9**	0.3	0.1
20	8.9	**76.9**	5.3	8.5	0.4	0.1
21	12.2	77.4	6.2	**3.9**	0.3	
22	4.3	**76.2**	6.0	13.0	0.4	
23	8.9	3.6	52.6	**34.3**	0.5	0.2
24	52.0	3.3	**39.0**	5.2	0.5	0.1
25	**45.9**	17.7	30.1	5.7	0.4	0.2
26	**95.6**	4.1			0.3	
27	**87.4**	7.6	4.1	0.3	0.6	
28	**91.8**	5.5	2.4	0.1	0.3	
29	7.8	**67.2**	12.6	11.8	0.6	
30	**99.4**	0.2	0.1	0.1	0.3	
31	6.6	5.6	**82.6**	4.7	0.6	
32	**56.5**	26.5	8.7	7.6	0.7	
33	38.5	15.0	26.6	**19.4**	0.5	
34	**77.7**	18.0	2.5	1.4	0.4	
35	10.0	**84.1**	5.3	0.3	0.3	
36	16.0	**61.9**	13.4	8.3	0.4	
37	10.2	20.9	**59.7**	8.2	0.8	0.1
38	**57.4**	18.6	12.3	11.0	0.5	0.1
39	10.2	**58.4**	13.6	16.9	0.9	0.1
40	47.3	8.2	**32.4**	11.4	0.6	
41	10.1	**85.1**	4.1	0.4	0.3	
42	29.0	**67.6**	1.0	1.9	0.5	
43	14.3	12.5	8.9	**63.3**	0.8	0.1
44	**89.4**	8.3	1.7	0.2	0.4	
45	**73.1**	14.0	8.4	3.8	0.7	
46	26.5	**33.7**	28.8	9.1	1.8	0.1
47	20.4	**79.1**	0.2	0.1	0.3	
48	17.2	4.6	2.0	**75.4**	0.7	0.1
49	20.4	**78.8**	0.1	0.4	0.3	0.1
50	16.0	10.6	1.7	**70.9**	0.7	0.1
51	**93.2**	1.3	4.4	0.7	0.3	0.1
52	**94.6**	0.9	2.3	2.0	0.3	
53	**88.2**	5.1	1.5	4.6	0.6	
54	**77.7**	10.9	9.2	1.7	0.4	
55	**2.5**	96.0	0.5	0.5	0.5	
56	**94.7**	0.4	4.1	0.5	0.3	
57	**95.4**	1.0	0.5	2.6	0.4	
58	**62.6**	21.2	5.3	10.2	0.6	0.1
59	**41.5**	24.7	19.0	13.9	1.0	
60	**83.1**	3.0	2.8	10.6	0.4	0.1
61	1.9	2.4	**94.7**	0.8	0.2	
62	35.6	3.0	1.0	**59.9**	0.3	0.1
63	23.7	21.8	**33.2**	19.9	1.2	0.1
64	4.0	8.7	26.4	**60.6**	0.4	
65	**67.8**	9.9	12.7	9.1	0.4	0.1
66	20.5	**32.3**	24.7	21.7	0.7	0.2
67	17.6	23.6	30.7	**27.5**	0.6	
68	33.2	3.6	9.2	**53.3**	0.6	0.1
69	**42.5**	22.7	17.2	17.3	0.4	
70	4.3	2.2	15.9	**77.1**	0.5	0.1

Bold values are the indices for the correct options, which also represent the ‘difficulty indices’
